# Prosocial rule breaking, ingroups and social norms: Parental decision‐making about COVID‐19 rule breaking in the UK

**DOI:** 10.1002/casp.2650

**Published:** 2022-09-06

**Authors:** Nicola Power, Lara Warmelink, Rebecca Wallace

**Affiliations:** ^1^ Department of Psychology, Fylde College Lancaster University Lancaster UK

**Keywords:** COVID‐19, ingroups, parenting, prosocial rule breaking, social norms

## Abstract

The British public generally adhered to COVID‐19‐related restrictions, but as the pandemic drew on, it became challenging for some populations. Parents with young children were identified as a vulnerable group. We collected rich, mixed‐methods survey data from 99 UK‐based parents (91 mothers) of children under 12, who described their lockdown transgressions. Household mixing was the most prevalent broken rule. Template analysis found that rule breaking was driven by ‘ingroup‐level’ prosocial motivations to protect the mental and social health of family and loved ones, and that parents were ‘engaged’ decision‐makers who underwent careful deliberation when deciding to break rules, making trade‐offs, bending rules, mitigating risks, reaching consensus, and reacting to perceived rule injustices. Cumulative link models found that the perceived reasonableness of rule violations was predicted by social norms. Rules were broken by parents not for antisocial reasons, but for ‘ingroup‐level’ prosocial reasons, linked to supporting loved ones.

## 1 INTRODUCTION

To combat the spread of COVID‐19, the British public entered COVID‐19‐related restrictions on 16 March 2020. Rules varied during this period, but have included the adoption of face coverings indoors, social distancing, bans on household mixing, the ‘rule of six’ (i.e., not meeting with more than six people), and ‘bubbles’ (i.e., only mixing with one other household if you live alone or require childcare). This led to changes in British society, with many adults working from home; children being unable to attend schools or nurseries; and health and social services being severely restricted.

Research has found that the public has adhered to lockdown restrictions even if they find them challenging. Duffy and Allington (2020) surveyed 2,250 UK citizens in April 2020 and found that up to 93% of adults reported abiding by lockdown restrictions completely or nearly all the time, with only 9% ‘resisting’ lockdown in some way. Adherence has in part been driven by people avoiding negative consequences, such as feeling personally at risk or fearful of COVID‐19 (Harper, Satchell, Fido, & Latzman, 2020; Smith et al., 2022) and avoiding fines (Chae & Park, 2020). Adherence has also been linked to shared group norms and community identification, leading to prosocial behaviour (Goldberg et al., 2020; Reicher & Drury, 2021; Stevenson, Wakefield, Felsner, Drury, & Costa, 2021). Prosocial behaviour aims to help another person or group, which may involve self‐sacrifice for no obvious gain (Penner et al., 2005); for example, abiding by restrictions when at a low personally perceived risk of severe disease.

## 2 ANTISOCIAL VERSUS PROSOCIAL RULE BREAKING

There has been a common (mis)perception that COVID‐19 rule breakers are motivated by anti‐social reasons. The term ‘pandemic fatigue’ has been used to describe the loss of motivation and impaired ability to adhere with lockdown restrictions (Michie, West, & Harvey, 2020). Linked to boredom (Boylan, Seli, Scholer, & Danckert., 2021), pandemic fatigue assumes that people tire of restrictions and begin to break rules for selfish reasons. The term ‘covidiots’ was coined to describe selfish rule breakers, who are deemed to be irresponsible members of society. Yet, this view of rule breaking is narrow and simplistic. Reicher and Drury (2021) argue that the portrayal of pandemic fatigue and the term ‘covidiots’ creates an unhelpful blame culture that distracts from the many structural issues that have forced some individuals to violate restrictions. It ignores that deviations from normative standards may be for prosocial reasons (Bilancini, Boncienelli, Capraro, Celadin, & Di Paolo, 2020; Spreitzer & Sonenshein, 2004).

Prosocial rule breaking is defined as volitional rule breaking in the interest of another (Morrison, 2006). It has been linked to positive deviance, wherein individuals diverge from norms with honourable intentions, such as breaking a rule at work to try and protect the reputation of their company (Spreitzer & Sonenshein, 2004). In the context of COVID‐19 restrictions, this may reflect breaking rules in the best interests of another person.

## 3 THEORETICAL DRIVERS OF COVID‐19 RESTRICTION ADHERENCE

The Theory of Planned Behaviour (Ajzen, 1991) states that behaviour is driven by intentions, which are themselves driven by attitudes, perceived behavioural control, and subjective norms. All three factors have been shown to affect COVID‐19 restriction adherence. Positive attitudes to COVID‐19 restrictions have been associated with greater adherence to restrictions (Azene et al., 2020). With regards to perceived control, Smith et al. (2022) showed that perceiving COVID‐19 measures as effective was associated with greater rule adherence. In terms of subjective norms, Goldberg et al. (2020) showed that perceiving friends and family as adhering to rules (i.e., a social norm) had a substantial positive influence on self‐reported adherence. Stevenson et al. (2021) also found that pre‐pandemic community identification was associated with increased in‐group helping and adherence to rules, suggesting a group‐level normative influence on behaviour.

Social identity theory (Tajfel & Turner, 1979) argues that individuals adopt the norms of those who they identify as being in their ingroup (Van Vugt & Hart, 2004). Research on collective behaviour during emergencies found that when individuals perceived a ‘common fate’ with others, it led to shared identity, greater solidarity, and cohesiveness (Drury, 2018). However, social norms might differ between higher and lower order ingroups, which can cause conflict (Penner et al., 2005). Khokhlova, Lamba, Bhatia, and Vinogradova (2021) reported that having faith in the government was associated with greater self‐reported adherence to COVID‐19 guidelines. Therefore, groups who perceive themselves to be unsupported by authorities and unfairly disadvantaged against may develop alternative group norms that reduce their adherence to preventative measures (Neville, Templeton, Smith, & Louis, 2021). If restrictions are perceived to be disadvantaging lower‐order ingroups (such as families, neighbourhoods, or socio‐economic groups), individuals might shift focus from ‘society level’ prosocial behaviours (e.g., adhering to restrictions) in favour of ‘ingroup‐level’ prosocial behaviours (e.g., violating restrictions to protect the family from reduced income or loneliness). Reicher and Stott (2020) warned that structural inequalities, a failure to help people cope with restrictions, and a move towards enforcement rather than promotion of rules could derail collective adherence. A greater understanding of the role of social normative standards is therefore needed.

## 4 THE PRESENT STUDY

This paper collected exploratory, in‐depth, mixed methods survey data to build a better understanding of rule breaking. We focussed on parents with young children as our chosen sample as parents have been identified as a vulnerable group for coping with lockdown restrictions due to their unstable financial circumstances, school closures and lack of support (Fontanesi et al., 2020), their greater risk of redundancy (Smith & McClosekey, 2020), and the removal of almost all ante‐ and post‐natal services (Davenport, Meyer, Meah, Strynadka, & Khurana, 2020). We collected qualitative accounts of people's rule breaking and their reasoning. We also gathered quantitative data on the perceived reasonableness of their rule breaking, social norms about rule breaking (measured by perceived rule breaking by others) and perceived risk of COVID‐19. The quantitative analysis used reasonableness ratings of rule breaking as the outcome variable, as this variable gives the greatest insight into people's social and moral perceptions of rule breaking. Our paper had three research questions:

1. What cognitions, motivations, and rationales do parents report for their decisions to violate restrictions?
2. What factors predict the perceived reasonableness of personal rule violations?
3. What factors predict the perceived reasonableness of others' rule violations?

This study was preregistered at https://osf.io/a3ndv.

## 5 METHOD

### 5.1 Participants

Participants were recruited using social media and snowball sampling. Participants were invited to participate if they had broken COVID‐19‐related restrictions at least once and were willing to discuss this anonymously. We targeted online groups aimed at parents with children under age 12 (e.g., closed parenting groups on Facebook). A total of 99 participants took part (91 females, 8 males; *M*_age_ = 34.09 years, *SD* = 5.26; 8 reported having had COVID‐19, 10 suspected they had had COVID‐19, and 81 reported not having had COVID‐19; see Data S1). We took a contextualised philosophical approach (King & Brooks, 2017) and used inductive thematic saturation (Saunders et al., 2018) to decide on sample size as our research question was a relativist exploration of parental lockdown transgressions, stopping data collection when no new themes were generated.

### 5.2 Materials

The survey was designed and distributed on Qualtrics from 18 January until 3 February 2021. This was during the third lockdown in England when the Delta variant led to high case numbers (highest 7‐day average on 18 January with 45,000) and many deaths (highest 7‐day average on 23 January with 1,248) (Financial Times, 2022).

#### 5.2.1 Lockdown violations

Participants were asked to provide open‐text responses to three qualitative questions discussing: (a) the lockdown rules they had ‘relaxed or bent’: (b) the main reasons for rule bending; and (c) the information sources they used to help make their choice.

#### 5.2.2 Self‐reasonableness data

Participants were asked to rate how reasonable they perceived their rule bending to be using a Likert scale from 1 (extremely reasonable) to 5 (extremely unreasonable).

#### 5.2.3 Other‐reasonableness data

Participants rated the reasonableness of other people's rule violations, based on eight scenarios adapted from a thread on Mumsnet (see Data S1).

#### 5.2.4 Social norms

We asked participants to estimate the percentage of parents they believed to be bending rules. Participants were asked to think about the rule they had broken and rate their likelihood (1–5 scale) of telling the following social groups: ‘parent(s)’; ‘close friend(s)’; ‘colleague(s)’; or ‘post on social media’.

#### 5.2.5 Perceived risk from COVID‐19

Participants were asked to rate how much risk they perceived from COVID‐19 on three items.

### 5.3 Data analyses

#### 5.3.1 Qualitative analysis of lockdown violations

The qualitative data was analysed using Template Analysis (King & Brooks, 2017). Template analysis is a type of thematic analysis wherein researchers combine a‐priori and inductive emergent themes to develop a template to explain the thematic relationships. We used simultaneous coding so quotes could be coded into multiple sub‐themes (Saldana, 2013). Our philosophical position was grounded in contextualism where we adopted a relativist ontological approach; we developed light‐tough a‐priori themes based on our interest in social norms and identities to inform our initial template, which we developed as we coded the data. We were specifically interested in understanding the contextualised experience of parents with young children, rather than seeking to generalise findings to non‐parent samples or parents with older children.

To support reflexivity and guard against bias, we built quality checks into our analysis using independent coding. The primary coder developed an initial template and shared it with a second coder, who then used the template to independently code the data and refine it in discussion with the primary coder. This template was presented to a third researcher, who provided ‘critical expertise’ and a challenge. Once a final template was derived, the coders returned to the data set and coded to consensus. The decision to code to consensus was based on the questionnaire nature of the data, where it was possible to identify if themes were present or absent for each participant. We were able to produce frequency counts for each theme to indicate the proportion of parents who self‐reported each theme. A further point on reflexivity is that the primary researcher is a parent to a young child. Positively, this meant that she could bring a contextualised understanding to the analysis through her experience, but this also risked bias in coding. To counter this, the second and third researchers on the team were not parents and independently coded the data as described.

#### 5.3.2 Predicting perceived reasonableness of own rule violations

We sought to identify whether the perceived reasonableness of one's own rule violations could be predicted by: (i) estimates of how many parents were breaking rules, (ii) openness to admit transgressions to other people, (iii) perceived risk from COVID‐19 and (iv) self‐reported rationales underpinning violation choices derived from the qualitative analysis. For (i), (ii) and (iii), predictors were added one at a time, however, for (iv), all subthemes under the same theme in the qualitative analysis were added at the same time.^1^ The ordinal package (Christensen, 2019) was used to create cumulative link models for the analysis. Different models were classified by their Akaike Information Criterion (AIC), with lower AICs reflecting better models. This analysis was different from the one proposed in our pre‐registration because these analyses are more suitable for the ordinal outcome variables.

#### 5.3.3 Predicting the perceived reasonableness of others' lockdown violations

We sought to identify whether the perceived reasonableness of others' lockdown violations (i.e., the scenarios) was predicted by the same variables as listed above. We also added the perceived reasonableness of one's own rule breaking behaviour to the model to explore whether the effect reported by Goldberg et al. (2020), that perception of others' behaviour affects our own, also occurs in the opposite direction: our perception of our own behaviour might affect our perception of others' behaviour. The same package as above was used to create cumulative link mixed models. For this analysis, the data was arranged in long format and a scenario variable was added.

## 6 RESULTS

All participants reported at least one transgression. Where participants reported multiple transgressions or provided multiple reasons for a transgression, all transgressions and reasons were coded.

### 6.1 Qualitative data on rule breaking

There were five rules that parents identified breaking. These were household mixing (83% of participants), meeting beyond the ‘rule of six/rule of two’ (13%), unnecessary travel (13%), exercising more than once per day (10%), and physical contact with someone outside the household (10%). The information sources they used to inform rule breaking included: (a) looking at official advice (62%); and (b) looking at the behaviour of others on social media (22%).

**FIGURE 1:**
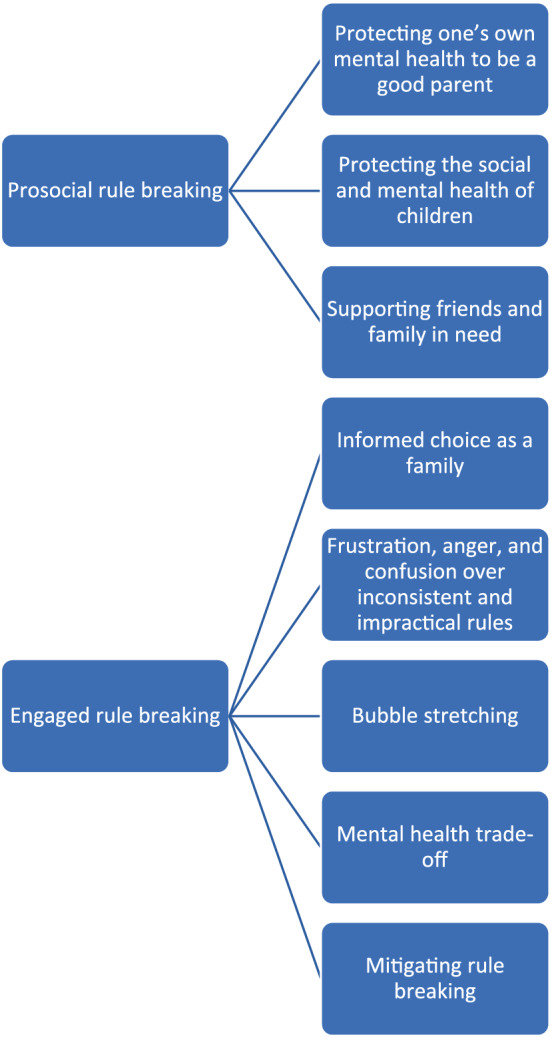
Cognitions underpinning lockdown rule breaking

We identified two main themes to reflect the cognitions underpinning parental rule breaking (Figure 1). These were: (a) prosocial rule breaking; and (b) ‘engaged’ rule breaking. The first theme reflected the motivation that participants described for rule breaking, and the second theme reflected how participants showed evidence of careful deliberation when making choices.

#### 6.1.1 Prosocial rule breaking

Rule breaking was driven by concerns about protecting the mental and social health of the family unit and loved ones. There were three sub‐themes: (a) protecting one's own mental health to be a good parent (50%); (b) protecting the social and mental health of children (45%); and (c) supporting friends and family in need (25%).

### 6.2 Protecting one's own mental health to be a good parent

Parents described a keen awareness of how their own mental health struggles during lockdown might impact upon their ability to be good parent. This sub‐theme was categorised as ‘prosocial’ as their concerns about mental health were rooted in concern over being able to care for their children. Parents described how they needed to break rules to care for their children: ‘for all our sanity, son with additional needs requires routine and working full time, my husband and I require childcare and the company of others to help us with mental health’(P6). Participants described relying on family members to give themselves respite and support their physical health: ‘we hadn't seen family for over a year and it was important for us to see them for the day and get some respite for ourselves after the move from abroad (I'm also currently 5 months pregnant)!’ (P26). Parents described how they lost the support networks they relied upon to care for their children: ‘During lockdown one, I was on maternity leave and found it so difficult. A newborn baby and a 3‐year‐old all day was draining and totally lonely’ (P23). They described their fear at being told to isolate and who was ‘allowed’ to help them look after their children when they needed support: ‘when the world locked down, I heard HIDE. Hide from the world with your baby, do not let anyone hold the baby, asking for help is not allowed, don't let your own mum hold your baby’ (P52).

Parents who had given birth just before or during lockdown described how lockdown fuelled post‐natal depression and difficulties caring for a new baby:

During the first lockdown I suffered post‐natal depression. My other half was still working full time and I was alone with the baby, sleep derived and mentally struggling. I had family members come round to help me cope. (P7).

### 6.3 Protecting the social and mental health of children

A second prosocial reason for breaking lockdown was to support their child's social and mental health. This included allowing children to mix with their friends because of loneliness: ‘my son experienced loneliness and lack of social network, not seeing other children in person for months has been devastating for him’ (P30), driven by a belief that children need to interact with others to stay healthy: ‘my children NEED to play with others for their mental wellbeing’ (P94). Concerns about the negative impact of limited social interaction were salient for parents with infants who were worried about their development:

I welcomed my first child 5 days before lockdown last March and I worry constantly about her social development – she's never been near another baby or anyone outside of my immediate family. I hope there'll be no long‐term impact on her. (P63)

Participants were motivated to break lockdown to ensure that their child was able to develop social bonds with family members, especially grandparents: ‘So my baby can get to know her family and so my family can build a bond with her. It has been especially difficult for grandparents to only see her through the window for her first 4 months’ (P25), and ensure they were not perceived as strangers: ‘I did not want my baby not to know who his grandparents were’ (P76).

### 6.4 Supporting friends and family in need

A final prosocial reason for violating lockdown rules was to support the social and mental health of adults outside one's own household. Participants described intergenerational caring responsibilities that they could not abandon during lockdown: ‘we felt that my mother‐in‐law and her partner are vulnerable to isolation and therefore we would have continued to visit regardless’ (P89). It also included providing support to vulnerable friends in need whose needs did not vanish despite the pandemic:

Providing childcare for the children of a friend with terminal cancer … and the reason to bend this rule, we thought, was perhaps just caring for vulnerable people who really needed it, and in a pandemic you sometimes just need to help others because other life is still happening. (P60)

Participants described a social responsibility to care for neighbours who lived alone: ‘visiting neighbour as she didn't have her usual groups and wasn't seeing anyone’ (P99). They described breaking rules to support the mental health of loved ones external to the household who missed their children: ‘because of how heart breaking I knew it was for them to be missing seeing their grandchild’ (P33).

#### 6.4.1 ‘Engaged’ rule breaking

The second main theme reflects how participants were engaged decision‐makers in deciding to violate lockdown rules, evidencing careful deliberation and clear rationales. There were five sub‐themes: (a) informed choice as a family (55%); (b) frustration, anger, and confusion over inconsistent and impractical rules (51%); (c) bubble‐stretching (37%); (d) mental health trade‐off (29%); and (e) mitigating rule breaking (20%).

### 6.5 Informed choice as a family

Participants described how their choice to break lockdown rules was based on informed consensus with their family network. Participants considered rules and statistics, but felt their choice was personal to their family and that they should not blindly follow the rules: ‘I knew facts and figures from the news and internet, but this was our personal choice as a family’ (P25). Their decision to violate lockdown restrictions involved trade‐offs between rules and the needs of their family: ‘we have read most of the available information (official and emerging via Twitter) and tried to reach a “least‐worst” option’ (P53). Participants who engaged in household mixing described a shared choice that was made with family members external to their household: ‘family discussion – all adults involved were the happiest with the arrangements we had’ (P67). Participants described personal decisions to bend rules for special occasions, such as celebrating birthdays: ‘we wanted to try and make the day special for my son, so we invited his two best friends over for a playdate’ (P54) and coping with bereavement: ‘we've had to bend the rules to plan a funeral, empty his house and grieve together’ (P76).

### 6.6 Frustration, anger, and confusion over inconsistent and impractical rules

Participants explained how they broke rules in reaction to perceived injustices by the government: ‘the government's swapping and changing of rules influenced me as I lost faith in their decisions and began to do what was best for my mental wellbeing’ (P8); and frustration at rule inconsistency: ‘Government advice was constantly changing and was no help’(P4). This was salient for new parents, who felt they were being undermined by contradictory rules on social mixing: ‘during the second lockdown 2,000 people were allowed to go and watch a football match but I couldn't attend a baby class??’(P36).

There was anger about the government's actions which motivated their rule breaking: ‘the government's handling has been abysmal. There was no consistency with rules, particularly between schools. The eat out to help out was the worst decision he made by a long stretch’ (P27), with participants describing unforgiving resentment at how they had been treated:

The conservative party and our government are a bunch of heartless, corrupt bastards and I hope and pray with every fibre of my being that the eventual outcome of this is that we will not have the Tories in power for a long, long, long time in the future. I have never been a conservative supporter, but I have never actively hated them as much as I do now (P35)

Participants questioned the reasoning behind rules: ‘It doesn't make sense to me when my two‐year‐old son needs to isolate because he has been exposed to someone who has tested positive in nursery, yet I do not and still need to go to work’ (P77), and described how they broke rules when they did not fit the reality of looking after children whilst working: ‘I had to have my sister and my mum care for my son whilst I worked’ (P84). This was especially problematic for key workers who worked irregular shifts: ‘As a response officer I cannot conduct my work from home and due to working a varied shift pattern, wrap about care from grandparents is needed’ (P8), who found it impossible to use one childcare bubble due to the unpredictability in their work patterns: ‘my whole family are key workers (mainly healthcare) who work different shift patterns and therefore unable to have assistance of just one grandparent household’ (P38).

### 6.7 Bubble stretching

Participants minimised rule breaking by describing it as a stretch of the support bubble definition. This included having multiple grandchildren in different households: ‘my parents have been looking after my one‐year‐old to allow me to go back to work. They also look after my nephew’ (P58), and creating a support bubble even if they did not technically meet the criteria: ‘whilst we did not qualify as a support bubble, the arrangement in terms of out‐of‐household contact was the same’ (P45). Parents used the term ‘support bubble’ to describe non‐essential childcare: ‘having my mother provide childcare has been a bending of the rules because, although she is in our childcare bubble, we have not strictly “needed” childcare as my husband has been furloughed’ (P57), and they socialised with their childcare bubble during handovers as they felt there was no additional risk: ‘often had a cup of tea with grandparents to get handover of children's activity’ (P44). The term ‘bubble’ was also used to justify household mixing in non‐childcare contexts: ‘we “bubbled” with friends (adult couple, no kids) for emergencies … our boiler has been broken for 3 weeks!’ (P53).

### 6.8 Mental health trade‐off

Participants described a deliberative trade‐off between rule adherence and its impact on their mental health: ‘me and my husband had long discussions about what risks we were going to take and balanced the physical risk with the benefit to our baby and mental health’ (P85). Participants portrayed an engaged thought process in which they knew they were breaking rules and were aware of the risks but decided that their mental health risk was greater: ‘You know you're breaking the rules, but your brain can't deal with any more isolation, so you risk it! Then go back to worrying about infecting people the day after’(P91). They described a crisis point that tipped the scales in their risk assessment: ‘I just hit a low and decided the risk for my mental health was far greater than the risk of COVID. My mum also felt this way’ (P33) and how the positives of social interaction outweighed the negatives: ‘our mental health influenced the decision. We knew it was wrong, but the consequences of seeing someone else, even for 5 minutes, outweighed the negative’. (P27).

### 6.9 Mitigating rule breaking

Parents engaged decision‐makers in rule breaking by mitigating risk in other ways, such as isolating before mixing with another household:

Since our return to the UK, we had been in 14 days self‐isolation … so although it was a bend of the rules, we felt safer in taking the risk as we believed the chances of contracting/passing on the virus was extremely low. (P28)

Participants also described getting a negative test result before mixing: ‘Although we know testing isn't 100% foolproof, the fact our family did have negative tests helped us feel easier with the decision’ (P28) and checking local infection rates: ‘we have always monitored the statistics throughout the pandemic and believed they were low at the time, so there was a minimal risk’ (P77).

### 6.10 Predicting the perceived reasonableness of own lockdown violations

**FIGURE 2:**
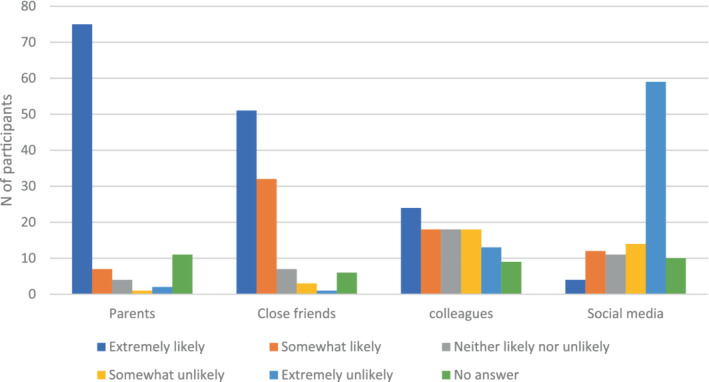
Frequencies of each response to the openness variables

Cumulative link models were used to investigate the predictors of participants' perceived reasonable of their rule breaking.^2^ The percentage of other parents that participants believed to be breaking rules was a significant predictor of their self‐reasonableness rating (estimate = −0.03, SE = 0.01, z‐value = −3.11, *p* = .002). The higher the perceived percentage of other parents breaking the rules, the more reasonable they perceive their personal rule breaking to be. The four openness variables showed a clear pattern of greater openness to people with whom the participants have closer relationships, with higher scores for parents, followed by close friends, colleagues and social media (Figure 2). Only ‘tell close friends’ improved the model (AIC from 217 to 212.7, LR stat [df = 5] = 14.09, 0.02). The coefficients showed that only ‘extremely likely to tell close friends’ had a significant estimate (estimate = −1.86, SE = 0.88, z = −2.11, *p* = .03): participants who are more likely to tell close friends about rule breaking thought their rule breaking was more reasonable.

Adding the perceived risk of COVID‐19 did not improve the model; neither the perceived risk that they would become infected (AIC = 212.7), nor the perceived risk that they would become ill (AIC = 215.9), nor the perceived risk that they would infect someone else if they became infected (AIC = 215.7) (all LR stats [DF = 5] < 9.85, all *p* > .08). None of the qualitative subthemes improved the self‐reasonableness model, nor were any significant coefficients found for any of the subthemes in any of the models (see Table 1).

**TABLE 1:** AICs for all models

Model	Predictors	AIC
1	% other parents adherence	217
2	% other parents adherence + tell close friends	212.7
3	% other parents adherence + tell close friends + risk infection	212.7
4	% other parents adherence + tell close friends + risk ill	215.9
5	% other parents adherence + tell close friends + risk infect other	215.7
6	% other parents adherence + tell close friends + rule broken	213.4
7	% other parents adherence + tell close friends + prosocial rule breaking	213
8	% other parents adherence + tell close friends + engaged rule breaking	217

### 6.11 Predicting the perceived reasonableness of others' lockdown violations

**FIGURE 3:**
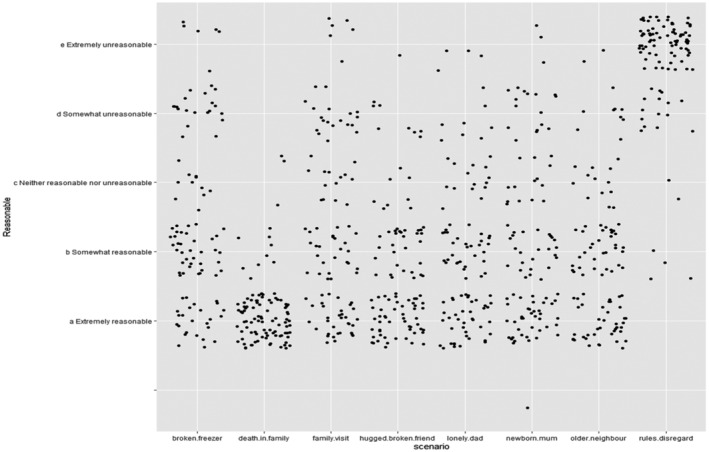
Jitter plot to show reasonableness ratings of different scenarios

A baseline model was created with only finished (a non‐significant predictor that indicated questionnaire completion) as a fixed factor and participant as a random factor. Adding scenario as a random predictor reduced the models' AIC from 2,317 to 1,748, which was a significant effect (LRstat [1] = 571.29, *p* < 02.2e‐16) (scenario Variance = 6.46). To explore this effect of scenario a jitter plot showing all responses was created (Figure 3). The scenarios varied in how reasonable the rule breaking in them was perceived to be. Notable are the ‘death in family’ scenario, which was perceived as extremely reasonable, and the ‘rules disregard’ scenario, which was perceived to be unreasonable.

Participants rating of self‐reasonableness significantly predicted their reasonableness rating of the scenarios (AIC reduced from 1,748 to 1,744; Lrstat [2] = 8.22, *p* = .02; coefficients in Table 2). The coefficient table (Table 2) shows that the effects are positive for all reasonableness ratings: the more reasonable participants perceived their own rule breaking to be, the more reasonable they rated the scenarios of other people's rule breaking behaviour. The estimate is the largest (and significant: z = 2.93, *p* = .003) between extremely reasonable and neither reasonable nor unreasonable. The effects for somewhat reasonable and somewhat unreasonable are not significant (somewhat reasonable: z = 1.91, *p* = .06; somewhat unreasonable: z = 1.26, *p* = .21). The ‘somewhat unreasonable’ results may well be due to the high standard error (0.91) and low sample size The four openness variables and the COVID‐19 risk factors were tested as possible predictors, but no significant predictors were found.

**TABLE 2:** Coefficients of reasonableness of own rule breaking on reasonableness of ratings

Coefficients	Estimate	Std. error	z‐value	Pr(>|z|)
Somewhat reasonable	0.73	0.38	1.91	0.06
Neither reasonable nor unreasonable	1.72	0.59	2.93	0.003
Somewhat unreasonable	1.15	0.91	1.26	0.21

*Note*: no‐one rated their own reasonableness as extremely unreasonable.

## 7 DISCUSSION

Our research suggests that COVID‐19 rule breaking was driven by ‘ingroup‐level’ prosocial motivations and by engaged, deliberative decision making. The most common rule that was broken was household mixing. We found that the perceived reasonableness of one's own rule breaking was predicted by social norms (supporting Goldberg et al., 2020), but not predicted by perceived risk (contrary to Harper et al., 2020). This supports theories (e.g., the theory of planned behaviour, the social identity theory) that rule breaking was influenced by perceptions about the behaviour of others. The perceived reasonableness of others' lockdown violations was found to be predicted by the perceived reasonableness of one's own behaviour, suggesting that individuals judge themselves in a similar way to others. This finding adds to the theories that state that other people's behaviour affects our own; own behaviour influences judgements about others as well. It is possible that this reciprocal relationship is due to an unmeasured confounding variable that mediates this relationship (e.g., attitudes towards COVID‐19 restrictions, attitudes towards rule breaking).

Participants in our study showed evidence of advanced moral understanding by acknowledging that they had broken lockdown rules whilst having a clear and justified rationale for why they chose to do so (Kohlberg, 2008). Although many parents might have adhered to society level prosocial norms in the early days of the pandemic, they had then shifted their normative standards towards a more nuanced moral consideration on the needs of their family and loved ones (‘ingroup‐level’ prosocial). This effect might superficially be like ‘pandemic fatigue’, but it is not driven by a shift from pro‐social to anti‐social behaviour. Rather the beneficiary of the prosocial behaviour is changing. Often this switch appears to be triggered by a crisis rather than by general fatigue: either an occurrence (e.g., a death) or a deterioration in the mental health or circumstances of either a loved one or the participants themselves.

Evidence for participants' careful deliberation about violations is found in the variability in parents' perceived reasonableness of violations in our scenarios, where the most antisocially motivated scenario (i.e., rules disregarded) was rated most negatively. Although participants had a clear reason for breaking rules, they were more likely to admit transgressions to parents and close friends than to colleagues or social media. This links to research on prosocial rule breaking, which found that when rules were broken ‘for the right reasons’, that they were still judged negatively by others (Dahling, Chau, Mayer, & Gregory, 2012). The decision to not be open about transgressions supports the notion of ‘engaged’ decision‐making (Reicher & Drury, 2021) and the awareness that their moral standards might not be shared by outgroups.

Parents described feelings of stress and isolation at being left to raise children alone and portrayed frustration, anger, and confusion over inconsistent and impractical rules. New parents described an intense feeling of discrimination where the rules meant that they were giving birth and looking after their newborn babies in isolation, whilst others were able to eat out in restaurants and attend football games. Reicher and Stott (2020) warned that structural inequalities can threaten adherence to lockdown restrictions as individuals perceive a disconnect between the rhetoric of ‘togetherness’ and the reality of how they are being treated. Injustice can weaken identification with higher order groups (i.e., the nation) to protect and promote the positive distinctiveness of one's ingroup (i.e., family/parents) who are being unfairly discriminated against by higher order social norms (Neville et al., 2021). Thus, the perceived fairness of rules is critical in motivating adherence and should be of the highest priority to rule makers.

### 7.1 Limitations and future directions

This paper adopted a mixed methods approach to explore and test the role of social norms in explaining rule violations for parents with young children. A limitation is that a mixed methods approach weakens our findings by trying to bridge disparate analytic approaches. Due to the exploratory nature of our paper, it was appropriate to qualitatively explore parents' experiences whilst also generating testable data. We had hoped that one way to bridge the qualitative and quantitative data would be by testing to see if our qualitative codes predicted reasonableness ratings, but we did not find any significant findings. This is possibly because themes were based on analysis of their self‐reported data; participants might not have reported themes themselves but would recognise them if asked explicitly. Future research could investigate this.

Another limitation of this paper was that it relied on self‐reported data that is open to bias. It is possible that parents attempted to justify their rule violations and felt that prosocial and engaged motivations would make them look better. The questionnaire was anonymous and the extent to which social desirability applies to self‐reported COVID‐19 regulations adherence has been questioned (Larsen, Nyrup, & Petersen, 2020). However, we cannot rule out that social desirability bias affected the data.

To study the reasoning behind lockdown violations, we recruited participants who had committed violations and were willing to give us information about them. There are many parents who adhered strictly to the rules, and that other parents will have violated restrictions but be unwilling to admit this. To what extent our findings on engaged deliberation extend to these individuals is unclear. Future qualitative research into why parents did adhere to lockdown would help clarify this.

Our data suggests that there might be interesting subgroup effects. Many parents of babies and toddlers mention the difficulties associated with very young children, suggesting that they feel older children would be easier to manage. The large number of women in our sample makes it hard to generalise the results to fathers. Future research should focus on collecting data from these subgroups to study these effects.

## 8 CONCLUSION

Using a mixed‐methods design, this paper has identified how violations of COVID‐19 restrictions by UK‐based parents were motivated by ‘ingroup‐level’ prosocial motivations to protect the mental and social health of the family unit and loved ones. Parents described being ‘engaged’ decision‐makers who carefully deliberated over rule breaking; making trade‐offs, bending rules, mitigating risks, reaching consensus, and reacting to perceived injustices. The perceived reasonableness of lockdown violations was predicted by social norms, and there was a relationship between the perceived reasonableness of one's own behaviour and that of others. This suggests a feeling of connectedness among the parenting population and supports our notion that normative standards were linked to ‘ingroup‐level’ prosocial norms. We argue that inadequate structural support for parents to cope with COVID‐19‐related restrictions influenced rule breaking. Parents were not motivated by a reckless disregard for society, but instead due to a shift in focus on the needs of social ingroups and a desire to take action to protect the family unit.

## CONFLICT OF INTEREST

There are no conflicts of interest to declare.

## ETHICS STATEMENT

This project was approved by the Faculty of Science and Technology Research Ethics Committee (FST20041) at Lancaster University, who abide by all ethics principles outlined by the BPS.

## Supporting information


**Data S1:** Supplementary Material

## Data Availability

The data that support the findings of this study are openly available in OSF at https://osf.io/74un6?view_only=f3d64e4716ab484f88b83a9daf90490b. Raw qualitative data has not been made available as per our ethics agreement with FSTREC at Lancaster University.

## Endnotes

^1^
Full R scripts including the data and notes are available at https://osf.io/74un6?view_only=f3d64e4716ab484f88b83a9daf90490b.

^2^
See https://osf.io/74un6?view_only=f3d64e4716ab484f88b83a9daf90490b for full script and data.
