# Supplementary material for: Prosocial rule breaking, ingroups and social norms: Parental decision‐making about COVID‐19 rule breaking in the UK
Source: J Community Appl Soc Psychol. 2022 Sep 6:10.1002/casp.2650. Online ahead of print. doi: 10.1002/casp.2650 (PMC9537870; doi:10.1002/casp.2650)

Demographic Information

	Number of participants	% Of participants
**Ethnicity**		
White	93	94%
Multiple Ethnicity	4	4%
Asian	1	1%
Other	1	1%
Total	99	100%
**Age of Participants children**		
Under 2 years	49	28%
Aged 2 to 4 years	57	33%
Aged 5 to 11 years	50	29%
Aged 12 to 18 years	11	6%
Over 18 years	6	4%
Total	173	100%
**Gender of Participants children**		
Female	83	48%
Male	90	52%
Total	173	100%
**Relationship Status**		
In a relationship - living separately	1	1%
In a relationship - living together	20	20%
Married	75	76%
Divorced/Separated	2	2%
Widowed	1	1%
Total	99	100%

Further characteristics of participant sample

	Average	Standard Deviation	Range
**Children per Participant**	1.75	0.747	1-4
**People in Household**	3.7	0.79	3-6

Scenario Details

Scenarios detailing real-life lockdown violations

Scenario Name	Brief Scenario Overview
Rules Disregarded	Has been seeing their partner who they don't live with every day, shopping for non-essential items, allowing their children to visit friends and believes the lockdown was a mistake.
Death in Family	Helped a friend out who had experienced a death in their family by looking after their child and does not feel guilty about doing so.
Heartbroken Friend	Hugged a devastated friend who had just lost their father to covid. Not proud of it but felt it was something they needed to do.
New-born Mum	Followed rules religiously but visited their mum after not seeing them since lockdown began. After seeing their mother every week for years, it was breaking the family's hearts and they couldn't handle not seeing her any longer.
Family Visit	Have followed lockdown so far but will be seeing family for genuine reasons. Both have been isolating separately for 6 weeks and are prepared to take the risk.
Older Neighbour	Helped their elderly neighbour whose hearing aid had broken and didn't have family that could visit. Believe chatting with him keeps him going. Doesn't feel in the least bit sorry.
Lonely Dad	Visits their dad every week as he is lonely and believes he would die happier if he passed covid onto him than if he was alone.
Broken Freezer	Sees parents regularly as using their parents' freezer and share a family business that requires close contact at times.

Jitter Plots

Jitter plot to indicate relationship between percentage of other parents perceived to be rule breaking and perceived reasonableness of own rule breaking behaviour

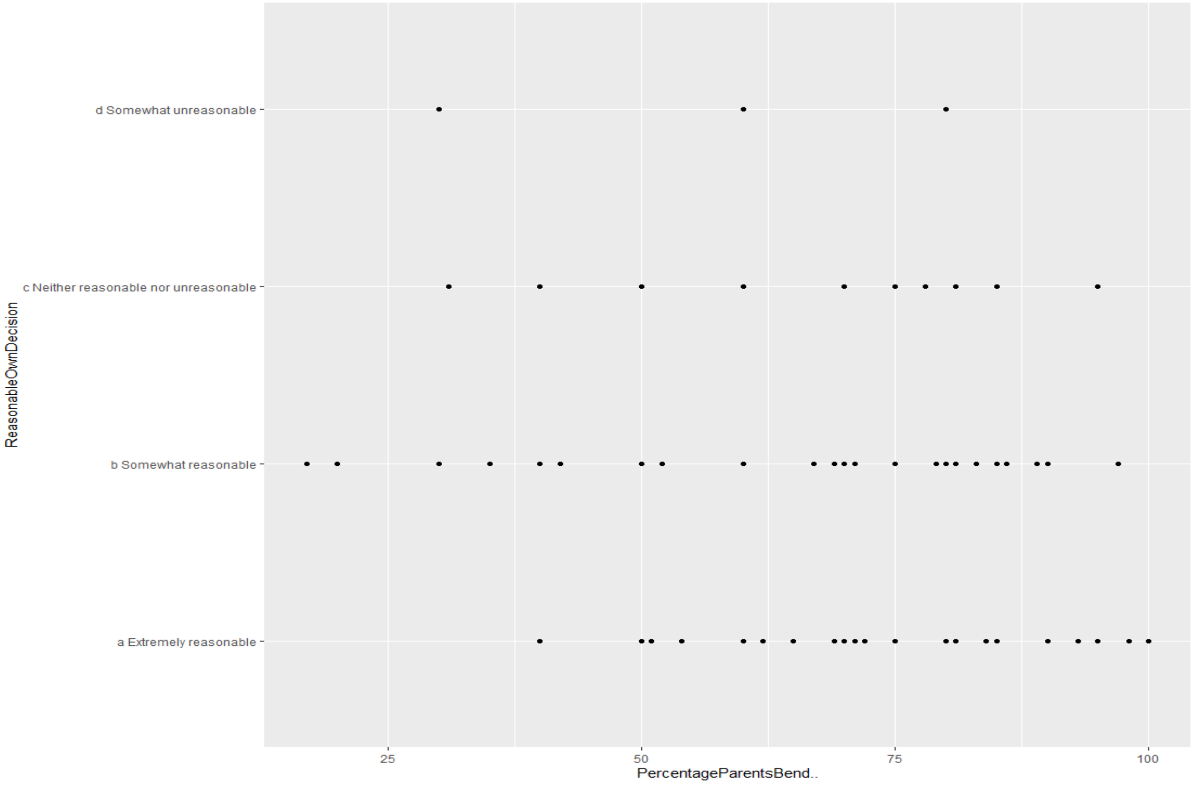

Jitter plot of own reasonableness as a predictor of others' reasonableness in scenarios

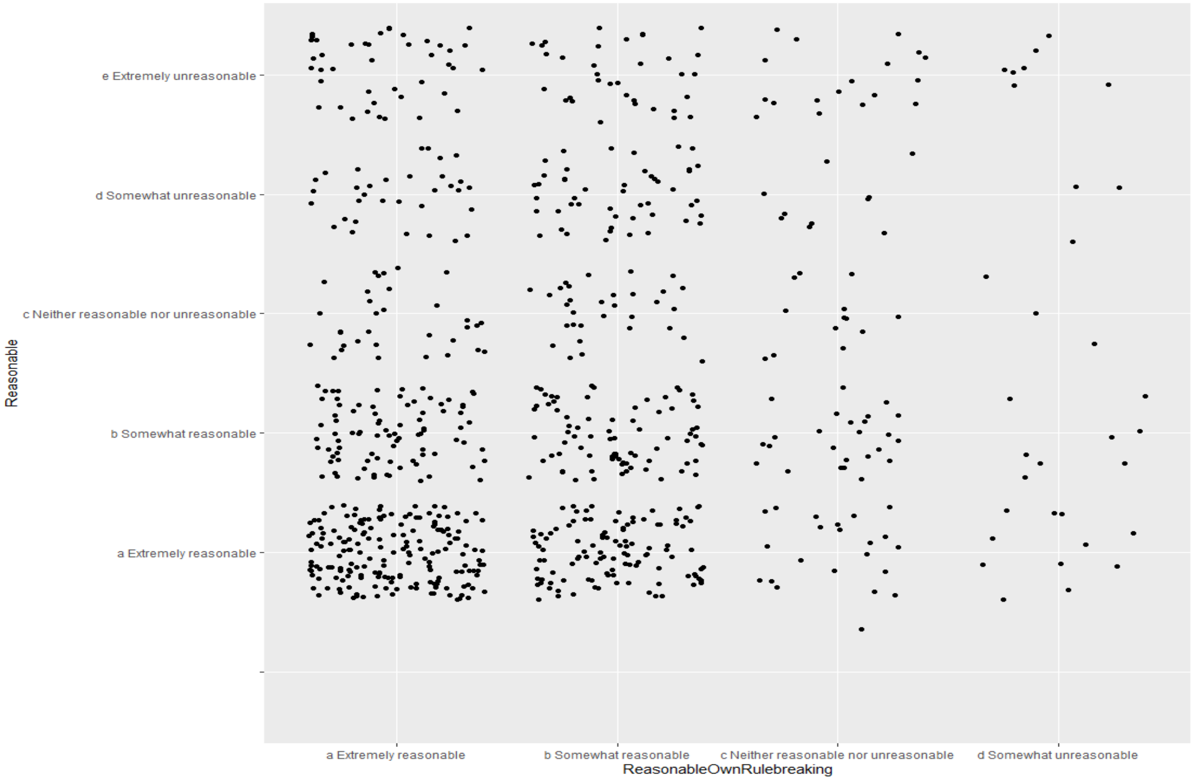

Supplement: Supplementary file 1 — Data S1: Supplementary Material [file CASP-9999-0-s001.pdf]
